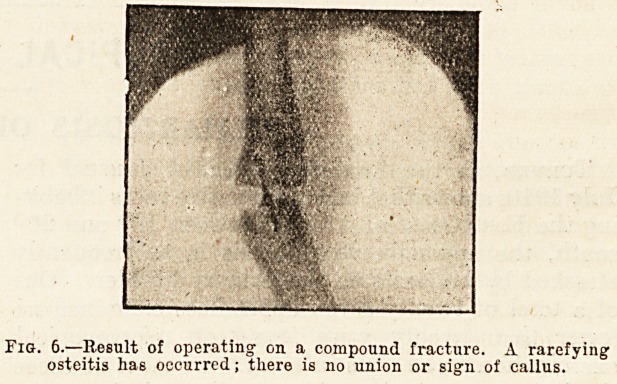# The Operative Treatment of Fractures

**Published:** 1911-01-21

**Authors:** 


### Surgery.

### THE OPERATIVE TREATMENT OF FRACTURES.

### III.—COMPOUND FRACTURES.

Turning from simple to compound fractures, we have an entirely different state of affairs. In a compound fracture we have the added risk of sepsis, and not by any means a slight risk. We approach the injury with the certain knowledge that micro-organisms have gained admittance to the wound. Our great aim, therefore, is to aid in every way the natural immunity of the part, so as to overcome these organisms and prevent the appearance of suppuration or marked inflammation in the wound. What, then, are the evil results that may follow suppuration in a compound fracture? In the first place comes death from pyæmia; this is seldom or never seen nowadays, but it must never be forgotten. Short of that, loss of the limb from a cellulitis or gangrene is a result that is still sometimes seen. But besides these more severe results there is the possibility of non-union. The factors described as causing non-union are many. But of cases seen clinically the large majority are due to inflammation at the site of fracture after a compound fracture. This may occur either with loss of bony substance from necrosis and sequestrum formation or without. In fact, in a certain number of compound fractures the skin wound apparently heals by primary union; there has been no sign of suppuration, and yet the bone does not join. This occurs far more frequently in compound fractures than in simple, and in many such cases the scar is keloid, a condition generally recognised now as being inflammatory.

Compound *v.* Simple Fractures.

It will be seen from the foregoing remarks that a comparison between a simple fracture cut down on by the surgeon under full aseptic precautions and an ordinary compound fracture is absurd. So also the treatment is different; when a foreign body is put into the one it is known that it will cause no trouble, that it will remain embedded in the tissues and never give rise to inflammation. But when such a thing is put in the other it is equally well known that if any inflammation should occur the foreign body will keep it up until it is removed; it is also known that there is a very good chance of inflammation occurring at the very site at which it is intended to put that body. If that were all it would be sufficient argument for not using screws and plates, wires, or other forms of internal splint in a compound fracture. But it is not all. Not only does the presence of the foreign body keep up the inflammation if it commence, but the putting of it in very greatly adds to the chance of such sepsis occurring and widening its area when it occurs. During the few short hours that have elapsed between the accident and the time of operation, an exudation has begun to shut off the infected area from the surrounding tissues, and above all from the broken bone. In putting in the foreign body this natural barrier is broken down, the muscles are raised from the periosteum, the periosteum from the bone, little pockets are formed in which bacterium-infected exudation may settle. More than this, holes are made in the bone, lowering its vitality and leaving free channels between the medulla and the outside. While all this is going on the natural repair of the part is lowered by the further injury to the tissues of the operation. In other words, to put an internal splint in a compound fracture is about the worst treatment possible. What are the results? Firstly, suppuration is far more likely to occur; it is a frequent occurrence for a compound fracture to suppurate; it is almost the rule for it to do so when a foreign body has been introduced. Secondly, when it occurs the results are far more serious, the bone is almost certainly involved, necrosis is very likely to occur with resulting loss of bone, sometimes an inch or more in length.

When this is the case, amputation generally has to be done, as the limb is useless. When this necrosis does not occur, prolonged suppuration,

Fig. 6.—Result of operating on a compound fracture. A rarefying osteitis has occurred; there is no union or sign of callus.

followed by a poor fibrous union which only becomes firm after eighteen months to two years may result.

Having discussed what should not be done in a compound fracture, we must now consider the positive side. How are we to obtain that perfect apposition which we have said must be the aim of the surgeon? The answer is that it cannot be done, and we must be content with the best possible result by the older methods of treatment. In the pre-antiseptic days compound fractures were most terrible injuries, with a high mortality. Antisepsis changed all that, and these accidents became but little more serious than a simple fracture. Asepsis has again changed the relative seriousness of the two; it has opened up a treatment for the one which is inapplicable both in theory and in practice to the other. Therefore we must again realise how much more serious is the latter injury, and not allow either ourselves or our patients to expect too good a result.

The Technique.

An anæsthetic should always be given, and the whole skin area thoroughly cleaned up with soap and water and antiseptic. The next point to consider is whether the wound should be opened up and the tissues underneath subjected to antiseptics? In most cases this is unnecessary, as the injury done to the tissues is as great as that done to the micro-organisms, and the power of repair is lowered. A simple dressing under anæsthesia and application of a splint in the best position possible is all that should be done. In other cases, however, the skin wound should be enlarged. When some clothing or dirt has been ground into the wound, when the skin has been stripped up from surrounding tissues, or where there is much blood-clot, the parts may be cleaned inside as well as out, small pieces of comminuted bone may also be removed. In all cases free drainage must be obtained.

This is all the operative treatment that should be done in these cases; the rest of the treatment is to obtain the best functional result possible short of what can be done by perfect apposition.

Two arguments are raised against this method of treatment and in favour of more extensive operations. First, a single case, or even two or three, is put forward in which the disasters indicated above have not occurred. The fact must not be considered on one or two cases, but on a large number. When the different methods of practice are observed over a long period of time it will be readily recognised that the results of putting foreign bodies in compound fractures are bad. It is as bad an argument to justify this treatment in compound fractures by one or two good results, as it is to condemn the treatment in simple fractures on a few good results without it.

The other argument is, "What could be done without? The position was so bad that it could not be got into anything like a good one without a plate." It may be admitted that sometimes in these cases this may seem to be so. But the surgeon would be better advised to stay his hand. In such a case the risk of sepsis is still more likely, and the plates only have to be taken out again soon after they have been put in.

Some operation for mal-union may be possible at a later stage, but to make the best of a bad job is all that can be done at the first.

To sum up, in advancing their ideals of the results of simple fractures, some have tried to bring up into the same line their ideals of compound fractures in the face of a pathological fact which makes this impossible. And that pathological fact is sepsis.

[We are indebted to Mr. W. Arbuthnot Lane for permission to use the photographs illustrating this series, which were taken from his cases.—Ed. The Hospital.]

## Figures and Tables

**Fig. 6. f1:**